# *Med14* cooperates with *brg1* in the differentiation of skeletogenic neural crest

**DOI:** 10.1186/s12861-015-0090-9

**Published:** 2015-11-09

**Authors:** Xin Lou, Jeffrey T. A. Burrows, Ian C. Scott

**Affiliations:** Model Animal Research Center, Nanjing University, 12 Xuefu Road, Nanjing, 210061 Jiangsu China; Program in Developmental and Stem Cell Biology, The Hospital for Sick Children, 686 Bay Street, Toronto, Ontario M5G 0A4 Canada; Department of Molecular Genetics, University of Toronto, 1 King‚s College Circle, Toronto, M5S 1A8 Ontario Canada; Heart & Stroke/Richard Lewar Centre of Excellence, University of Toronto, 101 College Street, Toronto, M5G 1L7 Ontario Canada

**Keywords:** Neural crest, Mediator complex, BAF complex, Brg1, Craniofacial defect, Med14, Jaw development

## Abstract

**Background:**

An intricate gene regulatory network drives neural crest migration and differentiation. How epigenetic regulators contribute to this process is just starting to be understood.

**Results:**

We found that mutation of *med14* or *brg1* in zebrafish embryos resulted in a cluster of neural crest cell-related defects. In *med14* or *brg1* mutants, neural crest cells that form the jaw skeleton were specified normally and migrated to target sites. However, defects in their subsequent terminal differentiation were evident. Transplantation experiments demonstrated that *med14* and *brg1* are required directly in neural crest cells. Analysis of *med14*; *brg1* double mutant embryos suggested the existence of a strong genetic interaction between members of the Mediator and BAF complexes.

**Conclusions:**

These results suggest a critical role for Mediator and BAF complex function in neural crest development, and may also clarify the nature of defects in some craniofacial abnormalities.

## Background

The neural crest lineage is a multi-potent, migratory population that is unique to vertebrate embryos and gives rise to a diversity of cell types including melanocytes, craniofacial cartilage and bone, smooth muscle, peripheral and enteric neurons and glia [1]. The specification, migration and differentiation of neural crest cells are tightly coordinated during development, with defects in these processes leading to a number of congenital diseases [2]. There is good evidence that during vertebrate embryogenesis a highly conserved neural crest gene regulatory network (GRN) orchestrates transcriptional events that are critical for various steps of neural crest development [3]. In addition to transcriptional regulation, mounting evidence supports a critical role for epigenetic regulation in neural crest development, most notably by controlling the timing of gene expression during this process [4].

Mediator is an evolutionarily conserved, multi-protein complex that is a key regulator of RNA polymerase II-mediated transcription [5]. In metazoan cells, multiple pathways that are responsible for homeostasis, cell growth and differentiation converge on Mediator through transcriptional activators and repressors that target one or more of the 30+ subunits of this complex [6]. Besides RNA polymerase II, Mediator interacts with and coordinates the action of numerous additional transcriptional regulators, including those acting at the level of chromatin remodeling [7, 8]. However, while *in vitro* data indicates the interaction between Mediator and chromatin remodelers, there is little *in vivo* data to support this hypothesis. Med14 is a subunit of Mediator that is essential for incorporation of the Tail module into Mediator [6, 9]. We have recently shown that Med14 plays an essential role in vertebrate embryogenesis and stem cell maintenance [10].

Working as a multi-subunit cellular machine that consumes ATP to modify DNA-histone contacts and modulate chromatin compaction, the BAF (BRG1/BRM-associated factors) complex plays a key role in many developmental processes by modulating gene expression. This further occurs via interaction of the BAF complex with transcription factors and other epigenetic readers at promoters and enhancers [11]. The BAF complex includes one of two core ATPases, Brm or Brg1, as well as a number of other subunits. *Brm* is dispensible for mouse development, whereas *Brg1* (*Smarca4*) is essential for broad aspects of embryogenesis [12, 13]. Differential inclusion of other subunit variants can give novel functions to the BAF complex in processes including neuronal development and cardiogenesis [11, 14, 15]. Reports have demonstrated a role for the Mediator complex in recruitment of the BAF complex to promoters or enhancers of target genes [16, 17], however no *in vivo* evidence for this genetic interaction and its importance exist to date. Defects in neural crest cell-derived tissues has been noted in *brg1* mutants [18] and recent work has shown that the BAF complex co-operates with CHD7 to orchestrate the expression of genes that regulate the migration of neural crest cells [19]. However, the mechanism underlying these roles in neural crest development has to date not been well characterized.

In the present study, we sought to determine the roles of *med14* and *brg1* during neural crest cells differentiation, and examine any possible genetic interactions. We found that *med14* mutant zebrafish embryos demonstrated multiple neural crest cell-related defects. Further analysis indicated that specification and early migration of neural crest cells occurred normally in *med14* mutants, with neural crest cells of the jaw subsequently failing to undergo terminal differentiation at their target sites. We further found that mutation of *brg1* also resulted in similar abnormalities. Analysis of *med14* and *brg1* double mutant embryos revealed strong genetic interactions between the Mediator and BAF complexes. Based on transplantation analysis, we found that both *med14* and *brg1* function in neural crest cells differentiation in a cell-autonomous fashion. Taken together, our results indicate that the BAF and Mediator complexes play essential and overlapping roles in the terminal steps of neural crest differentiation.

## Results

**Fig. 1:**
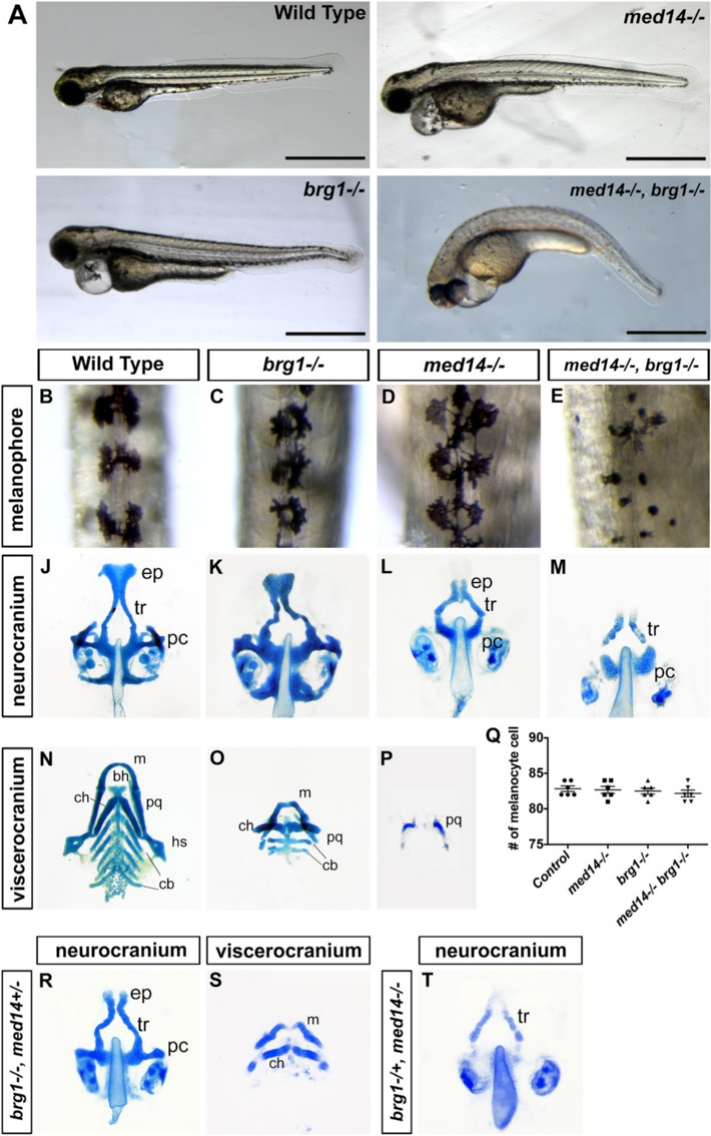
Multiple neural crest-derived phenotypes in mutant embryos. **a** Images of embryo morphology at 72 hpf. **b** to **e** Melanocyte defects in mutants at 48 hpf. Dorsal view with anterior to top. Scale bars, 1 mm. **j** to **p** and **r** to **t**) Alcian blue staining of cartilage reveals defects in neurocranium and viscerocranium formation in mutant embryos at 96 hpf. At least 15 embryos for each genotype were analyzed and representative samples are showed. Compared to single *brg1* or *med14* mutants, embryos bearing an additional copy of *med14* or *brg1* null allele show a more severe phenotype. Ventral view with anterior to the top. bh, basihyal; cb, ceratobranchial; ch, ceratohyal; ep, ethmoid plate; hs, hyosymplectic; m, Meckel’s cartilage; pc, parachordal plate; pq, palatoquadrate; tr, trabeculae. **q** Quantification of melanocyte number at 48 hpf on the trunks of control and mutant embryos. Twelve embryos for each genotype were counted. Error bars represent the SD

In unrelated studies, we noticed that zebrafish *log* (a null allele for *med14*) [10] and *young* (a null allele for *brg1*) [14] mutants shared a common array of deficiencies in heart, eye, pectoral fin and pigment cell development (Fig. 1a–d and data not shown). This similarity suggested overlapping or common functions for *med14* and *brg1*. To test this possibility, double mutants were generated. The *med14*; *brg1* double mutant embryos displayed a much more severe phenotype compare to single mutants, including a curved body axis, smaller eyes, severe heart edema and loss of pigment (Fig. 1a). To further investigate the role of *med14* and *brg1* in development, as well as possible functional interactions between the Mediator and BAF complexes, neural crest cell-derived tissues were analyzed in various mutant backgrounds. In *med14* and *brg1* single mutants, the melanin in melanocytes showed a less even and spiky distribution compared to controls; whereas in *med14; brg1* double mutants melanin distribution took on a small, rounded appearance (Fig. 1b–e). Quantification of melanocyte number on the dorsal surface of the trunk revealed no significant differences between controls and mutants (Fig. 1q, *P* = 0.94).

The development of the neurocranium and viscerocranium were next analyzed through Alcian blue staining at 96 h post-fertilization (hpf). In *brg1* mutants, the neurocranium was dismorphic, and the size of Meckel’s cartilage, ceratohyal and palatoquadrate on the viscerocranium were greatly reduced, with the last two branchial arches absent (Fig. 1j–k and n–o). In *med14* mutants, trabeculae formed, while most of the ethmoid plate and lateral parts of parachordal plate were not evident (Fig. 1l). Aside from relics of palatoquadrate, viscerocranium structures were absent in *med14* mutants (Fig. 1p). In *med14; brg1* double mutants, only posterior portions of trabeculae and parachordal plate were apparent, whereas viscerocranium was completely absent (Fig. 1m). It has been reported some posterior elements (part of trabeculae and parachodal plate) are derived from mesoderm [20]. As these elements remained in *med14; brg1* double mutants, this suggested *med14* and *brg1* are only required for neural crest-derived cartilage.

To further explore genetic interactions between *med14* and *brg1*, we examined cartilage defects in *brg1−/−*; *med14+/−* and *med14−/−*; *brg1+/−* embryos. In either case, further loss of one allele of *med14* or *brg1* in either *brg1* or *med14* null mutants resulted in more severe defects in facial cartilage formation as compared to single mutants (Fig. 1r–t). We also observed that *med14* and *brg1* mutants shared a common array of deficiencies in heart, eye and otic vesicle development. At 48 hpf, eye, otic vesicle and heart defects in *med14* or *brg1* mutant embryos were similarly exacerbated in *med14/brg1* double mutants (data not shown). Taken together, these data showed that zebrafish *med14* or *brg1* mutants displayed defects in neural crest-derived cells and tissues such like craniofacial cartilage and melanocyte. Furthermore, *med14; brg1* double mutants, or single mutants where one allele of the other gene was lost, displayed a more severe phenotype than single mutants, suggesting overlapping functions of the Mediator and BAF complexes.

**Fig. 2:**
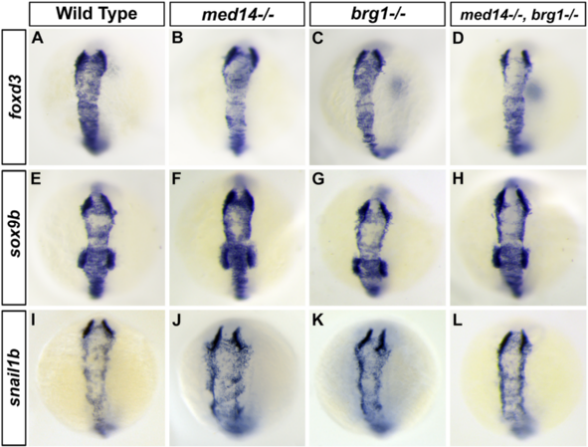
Neural crest cells are specified normally in mutant embryos. **a** to **l**: At 10 somite stage (14 hpf), expression of the neural crest specification markers *foxd3*, *sox9b* and *snail1b* were probed by RNA *in situ* hybridization. At least 20 embryos for each genotype were analyzed and representative samples are showed. Dorsal views with anterior to the top

**Fig. 3:**
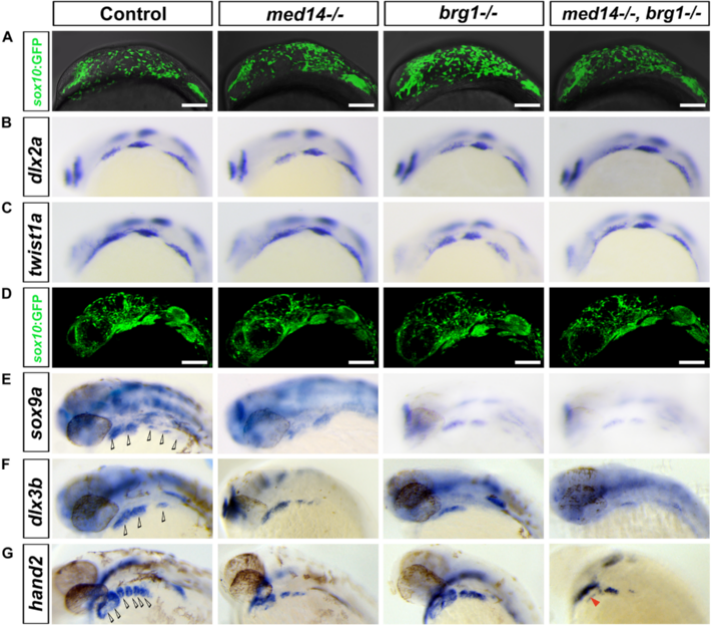
Defects in skeletogenic neural crest differentiation in the jaw forming area of *med14* and *brg1* mutant embryos. **a** to **d** Neural crest cells migrated to the oral ectoderm in both control and mutant embryos. **a** Snapshot of migrating neural crest cells (marked by *sox10:EGFP* transgene) in control and mutant embryos at 15 somite stage. **b** and **c** The migrating neural crest expressed *dlx2a* and *twist1a* at 18 somite stage. **d** At 24 hpf, neural crest cells reached the brachial arches forming region and condensed. **a** and **d**: lateral view with dorsal to top and anterior to left. **e** to **g** The mutants showed mis-expression of genes involved in mesenchymal condensations and chondrocyte differentiation. **b**, **c**, **e**, **f** and **g** RNA *in situ* hybridization is shown for expression of *dlx2a*, *tiwst1a*, *sox9*a, *dlx3b* and *hand2*. At least 15 embryos for each genotype were analyzed and representative samples are shown. *Hollow arrowheads* indicate pharyngeal arches; *red arrowhead* in **g** indicates the heart tube of embryo. Lateral views with anterior to the left. Scale bars, 100 um

As the phenotypes observed in *med14* and *brg1* mutants involved multiple tissues and developmental stages, we subsequently focused our analysis on jaw cartilage development to uncover the mechanisms of *med14* and *brg1* function in neural crest development. Neural crest specification was first analyzed via expression of *foxd3*, *sox9b* and *snail1b*, which are expressed in pre-migratory neural crest at the dorsal side of neural tube at 14 hpf [21]. No overt difference in expression was apparent between control and mutant embryos (Fig. 2a–l), indicating that neural crest cells were properly specified in *med14*, *brg1* and *med14; brg1* mutants. Subsequent migration of neural crest cells was studied via use of a neural crest-specific *sox10*:*EGFP* transgenic line [22]. Neural crest cells in all three mutant backgrounds dispersed and migrated around the eye and optic stalk to reach the oral ectoderm and formed primodia of branchial arches by 24 hpf; with the behavior of neural crest cells being similar between mutant and control embryos (Fig. 3a and d). In situ hybridization also showed that markers of migratory facial neural crest, *dlx2a* and *twist1a*, were expressed normally in mutants at 18 hpf (Fig. 3b–c).

**Fig. 4:**
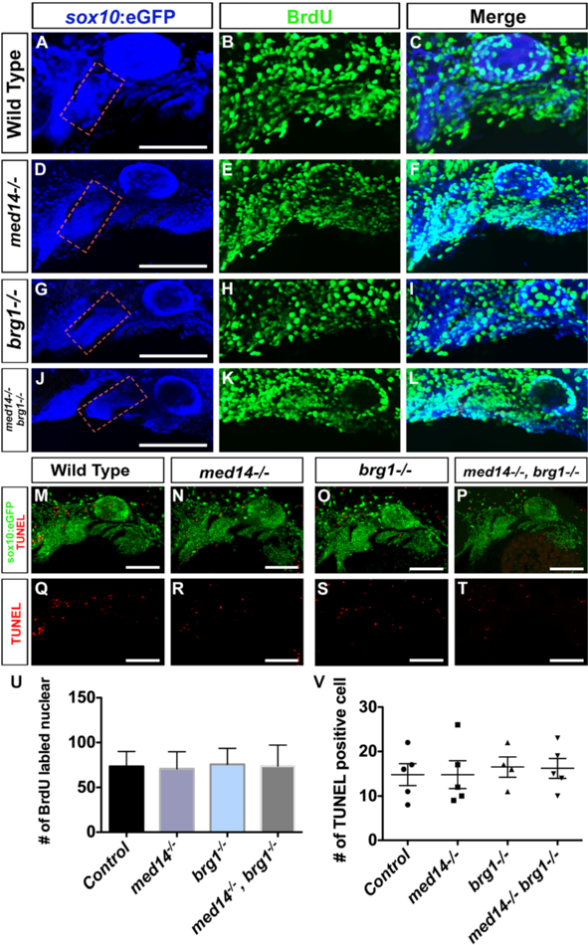
No apparent alterations in cell proliferation and cell death in arch-forming regions of mutant embryos. **a** to **l** Cell proliferation at 36 hpf was analyzed through BrdU incorporation. Neural crest cells were marked by *sox10:EGFP* transgene and revealed by immunostaining with anti-GFP antibody. Proliferating cells were marked by BrdU then revealed by immunostaining with anti-BrdU antibody. Lateral views with anterior to left. **m** to **t** Cell death at 36 hpf was analyzed through TUNEL assay. Lateral views with anterior to left. **u** Quantification of BrdU-positive cell in 2nd arch (*dotted squares* in **a**, **d**, **g** and **j**) in control and mutant embryos. Bars represent the SD. **v** Quantification of TUNEL positive cell in one side of pharyngeal arches in control and mutant embryos at 36 hpf. Bars represent the SD. Scale bars, 100 um

To determine if postmigratory cranial neural crest was properly maintained in these mutants, we examined expression of *sox9a*, *dlx3b* and *hand2*, which function in neural crest differentiation [23, 24], at 32 hpf. The expression of *sox9a* and *dlx3b* were both down-regulated in *med14* and *brg1* single mutants, and almost abolished in *med14; brg1* double mutants (Fig. 3e–f). At 30 hpf, *hand2* clearly marks rings of ventral neural crest in the pharyngeal arches (Fig. 3g). In *med14* and *brg1* mutant embryos, the expression of *hand2* was reduced (Fig. 3g). In *med14; brg1* double mutants, the expression was largely abolished except for in a small patch posterior to the eyes, which was maintained. Notably, *hand2* expression in the heart tube remained in double mutants, indicating that the regulation of *hand2* by *brg1* and *med14* is neural crest-specific (Fig. 3g). During jaw development, neural crest cells undergo extensive proliferation, so it is possible the defects observed in mutants were due to impaired cell proliferation or enhanced cell death. Cell proliferation and cell death were therefore analyzed at 36 hpf, however no obvious differences between control and mutants was observed (Fig. 4, *P* = 0.94 for cell proliferation and *P* = 0.95 for cell death). Taken together, this data suggests that in mutants neural crest cells are formed and migrate properly, but are subsequently unable to initiate a differentiation program towards becoming skeletogenic ectomesenchyme.

**Fig. 5:**
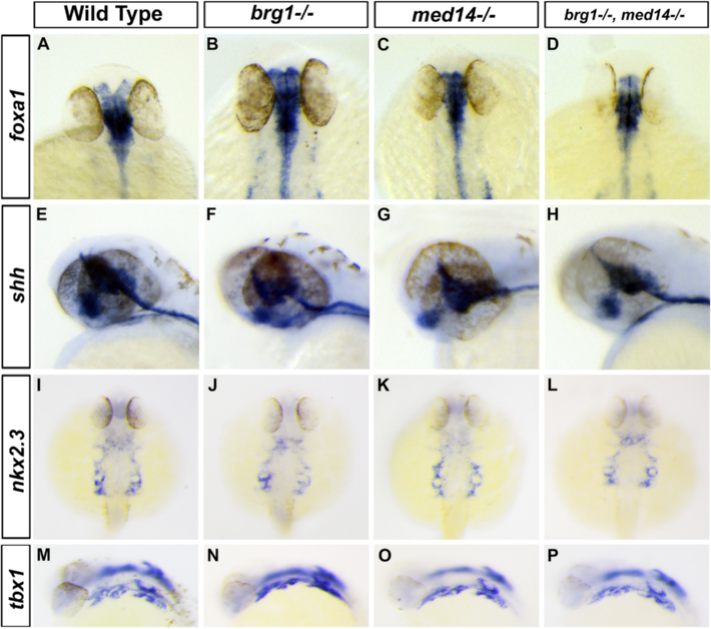
Mesoderm of pharyngeal arches and endodermal pouches are not affected in mutants. **a** to **d**: Expression of *foxa1* at 32 hpf in pharyngeal endoderm. **e** to **h**: The expression of *shh* in the stomodeum epithelium at 48 hpf. **i** to **l**: At 36 hpf, expression of the endodermal pouches marker *nkx2.3*. **m** to **p**: At 36 hpf, expression of the pharyngeal arches mesoderm marker *tbx1*. **a** to **d** and **i** to **l**, dorsal view with anterior to the top. **e** to **h** and **m** to **p**, lateral view with anterior to the left

Neural crest differentiation depends both on intrinsic gene regulatory programs and signals from the surrounding environment [25]. As such, deficiencies either in neural crest cells themselves or other tissues could be responsible for the defects we observed in *brg1* and *med14* mutants and compound mutants. As signals from the endoderm and notochord are indispensable for the migration and differentiation of pharyngeal neural crest cells [26, 27], we first analyzed expression of *foxa1* and *shh*, which are markers of these two tissues, at 30 hpf. Both genes showed apparently normal expression in mutants and compound mutants, suggesting that these tissues were not grossly abnormal (Fig. 5a–d and e–h). Since endodermal pouches play important role in patterning and differentiation of pharyngeal arches [28], we analyzed expression of *nkx2.3* and *tbx1*, which are expressed in both the pre-pouch endoderm and surrounding mesoderm [29]. At 32 hpf, expression of *nkx2.3* and *tbx1* appeared normal in both wild type and mutant embryos (Fig. 5 i to l and m to p), suggesting that induction of the endodermal pouches is not dependent on *med14* or *brg1* function.

**Fig. 6:**
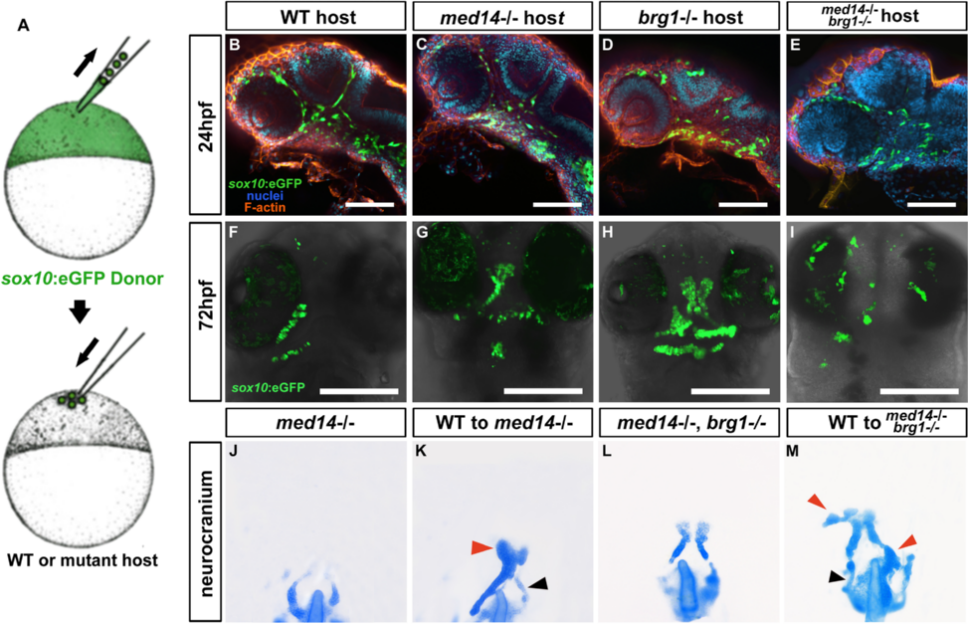
Wild type neural crest can contribute to jaw cartilage in mutant host. **a** Schematic diagram of the transplantation approach. Wild type *sox10:EGFP* transgenic donor cells are transplanted to the animal pole of wild type or mutant host embryos at 4 hpf. **b** to **e** At 24 hpf, donor-derived neural crest migration to the oral ectoderm is evident regardless of host genotype. Lateral views with anterior to the top. **f** to **i** At 72 hpf, donor-derived neural crest persistence and differentiation to cartilage is evident regardless of host genotype. Ventral views with anterior to the top. **j** to **m** At 72 hpf, cartilage staining reveals wild type donor-derived cells partially rescuing anterior neurocranium defects in *med14* and *med14*; *brg1* double mutant embryos. **g** and **k** represent images from the same embryo. *Red arrowheads* indicate cartilage derived from wild type donor cells; *black arrowheads* indicate host cell-derived cartilage. Dorsal views with anterior to the top. Scale bars, 100 um

To directly examine the cellular autonomy of *med14* and *brg1* function, we employed a transplantation approach. Cells were first taken from the animal pole of 4 hpf wild type *sox10*:*EGFP* donor embryos and transferred to the equivalent location in wild type or mutant 4 hpf host embryos (Fig. 6a). Based on their localization in the host embryo, a proportion of these naïve cells will normally take on a cranial neural crest fate in these experiments, as indicated by donor cell GFP expression. We observed that in all cases wild type donor GFP-positive neural crest cells in either wild type (*n* = 33) or mutant (*med14−/−*: *n* = 26; *brg1−/−*: *n* = 24; *med14*; *brg1* double mutant: *n* = 11) host embryos migrated to oral ectoderm and formed cartilage clusters, (Fig. 6f–i). Strikingly, as assayed by cartilage staining, we found that wild type donor cells could rescue anterior neurocranium cartilage phenotypes in *med14* (43 %, *n* = 34) and *med14; brg1* double mutant embryos (31 %, *n* = 14) (Fig. 6j–m). When *sox10:EGFP* activity was compared to cartilage staining results from the same embryos, we observed that anterior neurocranium cartilage entirely matched to GFP-positive clusters (Fig. 6g and k), strongly suggesting that wild type donor cells acted as the source of the cartilage.

**Fig. 7:**
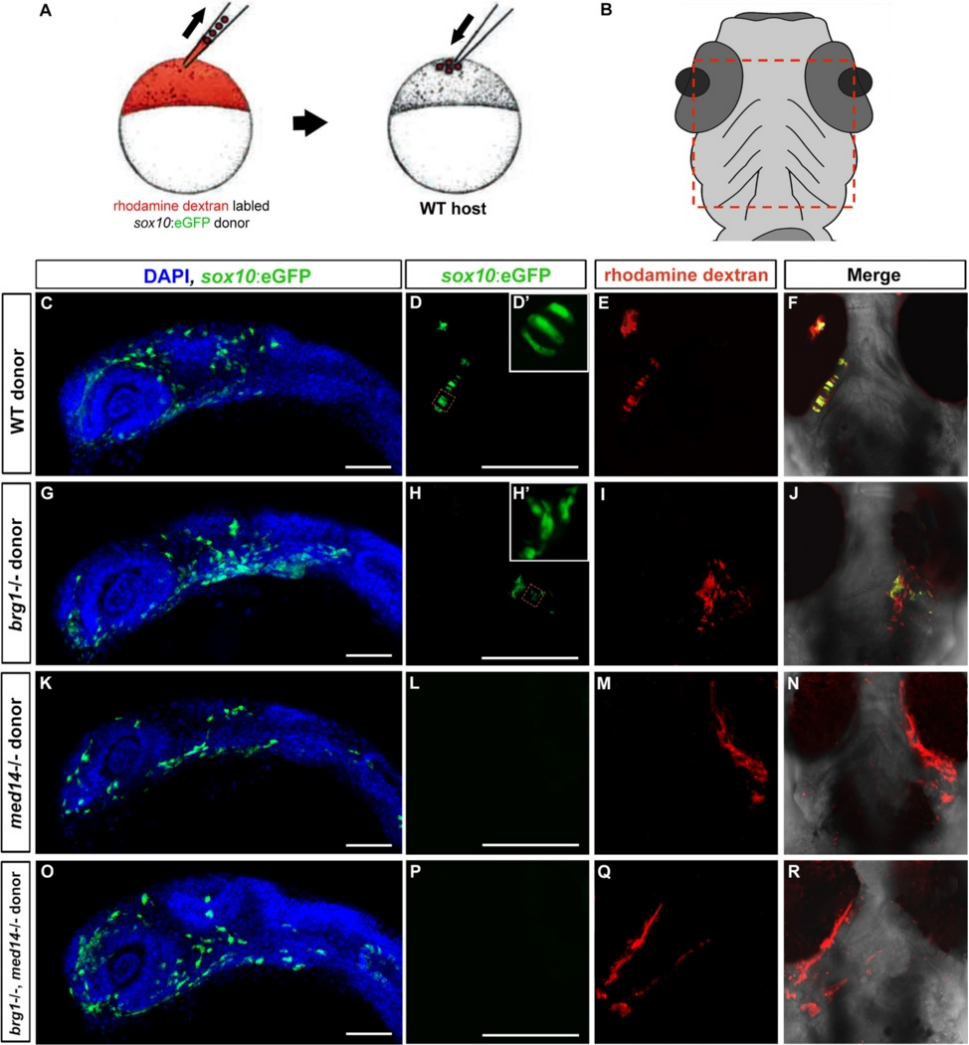
Cell autonomous requirement for *med14* and *brg1* in neural crest cells for cartilage differentiation. **a** Schematic diagram of transplantation approach. Donor (wild type or mutant) *sox10*:*EGFP* transgenic embryos were injected with rhodamine-dextran lineage tracer and transplanted to the animal pole of wild type host embryos at 4 hpf. **b** Diagram showed the region was imaged at 80 hpf. **c**, **g**, **k** and **o** At 24 hpf, donor-derived neural crest migration to the oral ectoderm is evident regardless of donor genotype. Later views with anterior to the top. **d**, **e** and **f**; **h**, **i** and **j**; **l**, **m** and **n**; **p**, **q** and **r** Donor cell contribution to cartilage assayed at 80 hpf. Ventral views with anterior to the top. D’ and H’: higher magnificent views of regions indicated by red squares in **b** and **e**. Scale bars, 100 um

To further examine the fate of *med14* and *brg1* mutant neural crest cells, transplantation experiments were next carried out in which wild type or mutant *sox10:EGFP* donor embryos were injected with rhodamine-dextran to allow observation of all donor cells (regardless of neural crest fate) (Fig. 7a). In wild type hosts, GFP-positive single or double *brg1* and *med14* mutant donor cells were observed that migrated to oral ectoderm at 24 hpf (Fig. 7 c, g, k and o). At 72 hpf, in 54 % of control (wild type donor, *n* = 44) transplantation experiments, donor cells were observed contributing to cartilage (Fig. 7d–f). In the case of *brg1* mutant donor cells, 11 % of transplants showed a contribution to cartilage (*n* = 31, Fig. 7h–j), with GFP-positive cells displaying abnormal cell shape (compared to the elongated cuboidal shape observed in control transplants (Fig. 7d and h). Strikingly, in none of the transplants where *med14* (*n* = 37) or *med14; brg1* (*n* = 21) mutant donor cells were used was contribution to cartilage noted (based on GFP expression). However, imaging of the donor cell lineage tracer (rhodamine-dextran) showed that mutant cells still survived in the jaw region of wild type hosts (Fig. 7l–n and p–r). These experiments indicated that both *med14* and *brg1* function cell autonomously in neural crest cells, to govern proper skeletogenic ectomesenchyme differentiation. Further, the persistence of the lineage label in mutant donor cells, despite the absence of cartilage formation, argues for a model where *med14* and *brg1* are required subsequent to migration of neural crest cells, where they are necessary to the initiation of chondrocyte differentiation. In the absence of this activity, neural crest differentiation to cartilage, and jaw development, is severely perturbed.

## Discussion

At different developmental stages, Mediator and BAF complexes influence a wide variety of biological processes. The nature of their activity is context dependent, and in many cases involves differential use of variant complex member subunits to confer different activities [11]. It will be interesting to investigate, specifically in the scenario of neural crest development, how these two complexes function in a tissue and developmental stage specific manner and how the genetic interactions revealed in this study are executed. An intriguing possibility is that subunit(s) of these two complexes act cooperatively to regulate the expression of genes critical for terminal differentiation of craniofacial neural crest cells. As neural crest contributes to a great diversity of additional cell types, and defects were observed in multiple neural crest-derived tissues in *med14* and *brg1* mutants, more specific analysis of defects in these cell types, and determining which genes are directly regulated by the Mediator and BAF complexes, will be of great interest. Further, while we have described defects in the maintenance of neural crest fate or terminal differentiation in this study, the actual fate of these cells is not clear. Or results suggest these cells are not lost via apoptosis. It will be of interest to determine if they adopt an alternative cell fate, and if so what this fate(s) and what mechanisms underlie this fate conversion may be.

In a previous report [19], knock down of *brg1* by morpholino injection in frog embryos led to defects in neural crest migration. Our data, however, suggests that the initial specification and early migration of cranial neural crest occurs normally even in severely affected *brg1; med14* double mutants, which is then followed by defects in skeletogenic neural crest differentiation from 30 hpf onwards. Furthermore, by using transplantation approaches, we have shown that *med14* and *brg1* act cell autonomously (in neural crest) to regulate differentiation of these cells. These discoveries reveal an unexpected mechanism in facial cartilage development, which has been previously ascribed to defects in migration of neural crest cells to the site of differentiation, and raises a possible mechanism underlying the symptoms of neural crest-related diseases, including CHARGE syndrome. It should be noted that these previous studies were largely based on use of morpholino oligonucleotides, whereas our current work uses genetic mutants for analysis. The *brg1* mutant used in this study [14] is a strong null allele, and phenocopies other described *brg1* mutants [18]. We have recently described three novel *med14* mutant alleles [10], with the *s231* allele used in this study having an equivalent phenotype to the *m628* allele that results in pronounced loss of transcript by nonsense-mediated decay. It is possible the maternally deposited (wild type) Brg1 and Med14 protein or transcript mask early effects on neural crest development in zebrafish mutants. However, as initial neural crest specification and migration appeared roughly normal even in double *brg1; med14* mutants, this seems unlikely.

Development of the neural crest is of exceptional medical importance, being affected in a large percentage of congenital defects. The results presented in this study and other recent publications show that the Mediator complex may contribute to a range of neural crest cell-related craniofacial abnormalities. The patients with CHARGE syndrome, a neural crest cell-related congenital anomaly, present a cluster of malformations in craniofacial, peripheral nervous system, ear, eye and heart [30]. Recent work has shown that the BAF complex orchestrates the development of neural crest cells via co-operating with *CHD7* [19], which is mutated in the majority of CHARGE syndrome cases [31]. Attempting to identify mutations affecting Mediator complex components in CHARGE syndrome and other neural crest-related patients will be helpful to further illustrate the molecular etiology of these syndromes. A more detailed analysis of mutant models of neural crest-derived craniofacial (and other) defects is also clearly warranted. Generation and analysis of *chd7* mutant zebrafish, for example, will be of great interest.

## Conclusions

In this study we used genetic tools to analyze the function and interaction of Mediator and BAF complexes on neural crest development. We found that mutation of *med14* or *brg1* in zebrafish embryos led a cluster of neural crest cell-related defects. Further analysis revealed additive genetic interactions between *med14* and *brg1*, resulting in more severe defects. These defects apparently arise not from an absence of neural crest at the site of cartilage development, but a failure of these cells to properly execute terminal differentiation into jaw cartilage elements. The genetic interaction between *brg1* and *med14* revealed in this present study indicates that Mediator complex function might act as an additional potential modifier in human congenital jaw defects.

## Methods

### Zebrafish lines and imaging

Zebrafish embryos were maintained and staged using standard techniques [32]. *Tg(sox10:EGFP)*^*ir937*^, *Tg(myl7:EGFP)*^*twu34*^ and *brg1*^*s481*^ fish have been previously described [22, 33, 34]. The *brg1*^*s481*^ allele was identified in a diploid ENU mutagenesis screen for mutations affecting endodermal organ morphogenesis [33]. A C-to-T base-pair change at position 754 in the *brg1* (*smarca4*) coding sequence creates a premature STOP codon at amino acid 252. The *med14*^*s231*^ allele was first recovered from a screen for cardiovascular mutants [35], and results in a premature STOP codon at amino acid 1200 in the Med14 coding sequence [10]. The *s231* allele acts as a null, phenocopying the *m628* allele in which mutant transcript levels are greatly reduced by nonsense-mediated decay. Imaging was performed using a Leica DFC320 camera on a Leica M205FA stereomicroscope. All confocal images were taken with Zeiss LSM510 confocal microscope.

### RNA probes transcription and RNA *in situ* hybridization

Transcription of DIG-labeled antisense RNA probes was performed using standard methods. RNA *in situ* hybridization (ISH) was carried out as previously described [36].

### Cartilage staining

Alcian Blue stainng was performed as previously described [37].

### Melanocyte counts

At 120 hpf, larvae were incubated in 10 mg/mL epinephrine (diluted in embryo media), anesthetized in tricaine and fixed in 4 % paraformaldehyde at room temperature for 2 h. Larvae were positioned on a spot plate using 3 % methyl cellulose to facilitate counting. Melanophore counts in all experiments included the dorsal (head, trunk and tail) lateral stripes.

### Cell proliferation and cell death assays

BrdU incorporation analysis and cell death assay were performed as previously described [38, 39]. Anti-GFP antibody from Abcam was used to label neural crest cells after HCl treatment. Cell death was assayed with a Cell Death Assay Detection Kit, POD (Roche), based on labeling of DNA strand breaks (TUNEL technology).

### Transplantation

Transplantation experiments were carried out as previously described [32, 40]. Donor and host embryos were subsequently kept in the same positions in a transplant mold until 48 hpf, when donor genotyping was carried out. For lineage tracing experiments, donor embryos were injected with 2 nl of 5 % tetramethylrhodamine dextran (10,000 MW, Molecular Probes) at the one-cell stage.

## Ethics approval

Zebrafish were housed and handled as per Canadian Council on Animal Care and Hospital for Sick Children Laboratory Animal Services guidelines.

## Abbreviations

BAF: BRG1/BRM-associated factors
dpf: days post-fertilization
GRN: gene regulatory network
hpf: hours post-fertilization
NC: neural crest
